# Digital Models as an Alternative to Plaster Casts in Assessment of Orthodontic Treatment Outcomes

**DOI:** 10.1155/2018/9819384

**Published:** 2018-06-12

**Authors:** Chung-Yan Vanessa Leung, Yanqi Yang, Chongshan Liao, Urban Hägg, Ricky Wing Kit Wong, Colman McGrath, Min Gu

**Affiliations:** ^1^Orthodontics, Faculty of Dentistry, The University of Hong Kong, 34 Hospital Road, Hong Kong; ^2^Department of Dentistry and Maxillofacial Surgery Cleft Center (Craniofacial Orthodontics), United Christian Hospital, 130 Hip Wo Street, Kwun Tong, Kowloon, Hong Kong; ^3^Dental Public Health, Faculty of Dentistry, The University of Hong Kong, 34 Hospital Road, Hong Kong

## Abstract

**Objective:**

This study aimed to compare the use of digital models and plaster casts in assessing the improvement in occlusion following orthodontic treatment.

**Materials and Methods:**

Digital models and plaster casts of 39 consecutive patients at pre- and posttreatment stages were obtained and assessed using the Peer Assessment Rating (PAR) index and the Index of Complexity and Treatment Need (ICON). PAR and ICON scores were compared at individual and group levels. Categorization of improvement level was compared using Kappa (*κ*) statistics.

**Results:**

There was no significant difference in neither PAR scores (*p* > 0.05) nor ICON scores (*p *> 0.05) between digital and plaster cast assessments. The Intraclass Correlation Coefficient (ICC) values for changes in PAR and ICON scores were excellent (ICC > 0.80). Agreement of ratings of occlusal improvement level between digital and plaster model assessments was 0.83 (*κ*) for PAR and 0.59 (*κ*) for ICON, respectively.

**Conclusion:**

The study supported the use of digital models as an alternative to plaster casts when assessing changes in occlusion at the ‘individual patient' level using ICON or PAR. However, it could not fully support digital models as an alternate to plaster casts at ‘the group level' (as in the case of clinical audit/research).

## 1. Introduction

There has been considerable development in digital models in orthodontics, which have obvious advantages in terms of storage space and ease of transfer and overcome the physical damage and loss problems associated with conventional plaster models [1]. On the other hand, plaster casts are also essential for use in orthodontic diagnosis and treatment planning, allowing us to evaluate treatment need and treatment outcome [2].

Despite the array of commercial companies providing digital models, studies have observed good agreement between digital and plaster models in the assessment of arch length; tooth size, overbite and overjet; and various intra-arch and interarch relationships.[2–4] Comparisons of Bolton Ratios between digital and plaster models have demonstrated clinically insignificant results between the two [5–7].

Potentially digital models may also be useful in assessing orthodontic treatment need and treatment outcomes. The Peer Assessment Rating (PAR) is a score to assess various occlusal traits making up a malocclusion. The individual scores are summed to obtain an overall total, representing the degree a case deviates from normal alignment and occlusion, which means the score of zero would indicate good alignment and higher scores indicating increased levels of irregularity [8]. Assessments comparing pretreatment PAR scores between digital and plaster models have shown substantial agreement [5, 9].

The Index of Complexity and Treatment Need (ICON), which comprises five weighted measurements, is based on the subjective judgments of 97 orthodontists from nine countries on 240 initial and 98 treated models [10]. Previous studies have reported no statistical difference in overall ICON scores between plaster and digital models from pre- and posttreatment assessment [11].

However, further study is required to support or refute such claims and to consider the level of agreement in a more comprehensive manner, i.e., considering not only the magnitude of change in scores but also agreement in the level of improvement obtained. This study aims to compare the use of digital models and conventional plaster models in assessing occlusal improvements by both PAR and ICON.

## 2. Materials and Methods

### 2.1. Study Sample

Prior to the study a sample size calculation was conducted based on the null hypothesis of good or excellent agreement of 0.7, with *α* at 0.05 and *β* at 0.2 (80% power), a sample size of 35 was proposed. Allowing for potential artefact anomalies, an additional 10% recruitment was planned. Eventually, 39 consecutive subjects were recruited from 2008 to 2012 in the Faculty of Dentistry of the University of Hong Kong. Pre- and posttreatment plaster casts and digital models (O3DM®) were obtained (78 sets of both) for these subjects.

### 2.2. Data Collection

Pretreatment and posttreatment models were assessed on both plaster casts and digital models. This was undertaken by a trained and calibrated examiner in the use of PAR and ICON indices. A digital calliper (Shanghai Taihai Congliang Ju Co., Ltd., Shanghai, China) was used to obtain the measurements from the plaster casts, to the nearest 0.01mm. For measurements of overjet and overbite, a standard ruler was used, and measurements were obtained to the nearest 0.1mm. Digital models were assessed using O3DM's designated software (www.o3dm.com/moderndentallab) to measure the individual components of PAR and ICON. All components were measured to the nearest 0.01mm.

Assessments of PAR were made according to the methods described by Richmond et al. [12] The five components of PAR include assessment of upper and lower anterior segment crowding, buccal occlusion, overjet, overbite, and centreline. Pretreatment and posttreatment plaster casts were measured according to the PAR index conventions and guidelines. The same counterpart digital models were assessed using the O3DM's own computer software. Each of the individual components was weighted accordingly to prescribed rubric, and the overall weighted scores were used for comparison.

The ICON assessment was conducted according to the methods and criteria described by Daniels and Richmond [10]. The five components of ICON include assessment of aesthetic assessment; upper arch crowding or spacing; crossbite; incisor openbite or overbite; and buccal segment anteroposterior relationship. Plaster casts and digital models were assessed and weighted, with the overall weighted scores being used for comparison.

### 2.3. Data Analyses

PAR and ICON scores of pre- and posttreatment digital and plaster models were derived. In addition, improvement in PAR and ICON scores was categorized according to outcome assessment criteria. [10, 12] Intraexaminer reliability (method error assessment) was calculated by using Dahlberg's formula [13], which was conducted by assessing on a random sample of 10% of both the plaster and digital models blind of the original assessments.

Agreement between digital and plaster models was examined using several analytical strategies. First, the mean directional difference between digital and plaster model PAR and ICON scores was calculated (plaster minus digital score). Then a test was performed to evaluate whether the mean of the directional difference was significantly different from zero. A mean directional difference significantly different from zero provides evidence of systemic bias between plaster and digital models. To examine systematic bias, effect sizes were calculated by dividing the mean difference score by the standard deviation of the difference score [14]. Secondly, the mean absolute difference was calculated using Wilcoxon signed-rank test. In contrast to the directional difference, the absolute difference ignores the positive and negative signs of the difference between plaster and digital models. Third, the Intraclass Correlation Coefficient (ICC) values between the digital and plaster casts were computed. The ICC is an appropriate measure of agreement as it corrects correlation for systematic differences and provides an unbiased estimate of agreement [15]. Following on, agreement between categorization of improvement in occlusion was determined using Kappa (*κ*) statistics [16].

## 3. Results

The method error for plaster casts for PAR and ICON was 3.15 and 3.4, respectively (*p* > 0.05); for digital models this was 2.6 and 4.4, respectively (*p* > 0.05).

PAR agreement results are presented in Table 1. There was no significant difference in neither pretreatment nor posttreatment plaster and digital model assessments based on the Wilcoxon signed-rank test (*p* > 0.05). The standardized difference of pretreatment models was < 0.30 and < 0.10 for the posttreatment models. Furthermore, there was no significant difference in the magnitude of change in PAR assessments obtained from plaster compared to digital models (*p* > 0.05); standardized difference < 0.30. The mean absolute difference in pre- and posttreatment assessments for both plaster and digital models was < 2.0 and for changes in PAR scores between plaster and digital models was < 2.0. The ICC values for pretreatment models were 0.989 (95% CI, 0.980 to 0.994) and for posttreatment models were 0.982 (95% CI, 0.966 to 0.991) and 0.988 (95% CI, 0.978 to 0.994) for changes in PAR scores. Agreement of the categorization of improvement in occlusion with respect to PAR scores was 0.83 (*κ*) (Table 2).

ICON agreement results are presented in Table 3. There was no significant difference in neither pretreatment nor posttreatment plaster and digital model assessments (*p* > 0.05). The standardized difference of pre- and posttreatment models was < 0.2. Furthermore, there was no significant difference in the magnitude of change in ICON assessments obtained from plaster compared to digital models (*p* > 0.05); standardized difference was < 0.10. The mean absolute difference in pretreatment models was < 10.0 and for posttreatment assessments was < 5.0 and for changes in ICON scores between plaster and digital models was <10.0. The ICC values for pretreatment models were 0.903 (95% CI, 0.817 to 0.949) and for posttreatment models were 0.859 (95% CI, 0.733 to 0.926) and 0.922 (95% CI, 0.852 to 0.959) for changes in ICON scores. Agreement of the categorization of improvement in occlusion with respect to ICON scores was 0.59 (*κ*) (Table 4).

## 4. Discussion

A number of analytical strategies were employed to provide a comprehensive assessment of agreement of orthodontic treatment outcomes between digital and plaster models using two of the most commonly used indices—PAR and ICON [10, 12]. The present study found that there was no significant difference in the directional difference between the digital and plaster models with respect to pretreatment, posttreatment, and change in scores. This concurs with the findings of others [5, 9, 11]. The present study also found that the standardized differences, as an indicator of systematic bias, was generally negligible (< 0.20) but with respect to agreement in changes, PAR scores was somewhat larger; although it can still be considered as a small ‘bias' [17]. These findings suggest that in assessment of agreement between digital and plaster models it is important to consider not simply agreement of pre- and/or posttreatment overall scores but also of ‘the change' in scores which is additional information that this study provides and has clinical relevance. Absolute difference values provide an insight into agreement irrespective of direction (ignoring the positive and negative change); the findings indicated that the absolute difference were generally small expect for changes in ICON scores (mean 9.13). Given that ICON score can range from 7 to 140, a difference of 9 constitutes approximately 7% difference that in itself may have no clinical significance.

There was no significant difference in categorization of improvement level in PAR or ICON scores obtained from digital and plaster models; Kappa values could be interpreted as ‘excellent' for PAR at 0.83, but only ‘moderate' for ICON at 0.59 [18]. This slight difference may be the result from a more stringent standard of ICON than PAR in the ‘greatly improved' category [19]. Therefore, at the group level, while there was generally good agreement for both PAR and ICON scores, variations in some situation the level of agreement were less than ideal, i.e., systematic bias (ICON). This may have implications with respect to clinical audit as well as research that rely on analyses of groups of patients.

## 5. Conclusions

In conclusion, findings from this study demonstrated that there are acceptable levels of agreement between digital and plaster models in the assessment of treatment outcomes. This study supports the notion that when assessing at the ‘individual patient' level digital models can be used as an alternate to plaster models which has obvious clinical in-practice implications. However, it could not fully support digital models as an alternate to plaster casts at ‘the group level' (as in the case of clinical audit/research).

## Figures and Tables

**Table 1:** Agreement between digital models and plaster casts in assessment of PAR.

	Weighted score			Directional difference		Standardize difference*∗*	Absolute difference		*p* value	ICC	95% Confidence Interval
	Mean	SD	Median	Mean	SD		Mean	SD			
Pre-treatment plaster cast	31.87	12.11	33	0.69	2.4	0.289	1.41	2.05	0.089	0.989	0.980-0.994
Pre-treatment digital model	31.18	11.87	32								
Post-treatment plaster cast	3.23	5.44	1	-0.08	1.35	-0.057	0.69	1.15	0.263	0.982	0.966-0.991
Post-treatment digital model	3.31	4.54	2								
Treatment change in plaster cast	-28.64	13.74	-30	-0.77	2.8	-0.275	1.9	2.17	0.141	0.988	0.978-0.994
Treatment change in digital model	-27.87	13.09	-30								

*∗*Standardized difference, D = mean directional difference/standard deviation of directional differences (D≦0.2, small; 0.2<D≦0.5, moderate; and D≦0.8, large).

**Table 2:** Agreement of the categorization of improvement in occlusion with respect to PAR scores.

	Improvement scale			Kappa value
	Worse-No Difference	Improved	Greatly Improved	
Improvement in plaster cast	5.13 % (2)	25.64 % (10)	69.23 % (27)	0.834
Improvement in digital model	5.13 % (2)	28.21 % (11)	66.67 % (26)	

**Table 3:** Agreement between digital models and plaster casts in assessment of ICON.

	Weighted score			Directional difference		Standardize difference*∗*	Absolute difference		*p* value	ICC	95% Confidence Interval
	Mean	SD	Median	Mean	SD		Mean	SD			
Pre-treatment plaster cast	57.05	20.21	58	-1.23	11.52	-0.107	9.08	7.04	0.561	0.903	0.817-0.949
Pre-treatment digital model	58.28	18.29	59								
Post-treatment plaster cast	12.97	6.96	13	-0.97	5.50	-0.177	3.54	4.29	0.303	0.859	0.733-0.926
Post-treatment digital model	13.95	8.64	13								
Treatment change in plaster cast	-44.08	22.05	-47	0.26	11.92	0.022	9.13	7.53	0.838	0.922	0.852-0.959
Treatment change in digital model	-44.33	21.7	-47								

*∗*Standardized difference, D = mean directional difference/standard deviation of directional differences (D≦0.2, small; 0.2<D≦0.5, moderate; and D≦0.8, large).

**Table 4:** Agreement of the categorization of improvement in occlusion with respect to ICON scores.

	Improvement scale					Kappa value
	Worse-No difference	Minimally improved	Moderately improved	Substantially improved	Greatly improved	
Improvement in plaster cast	2.56 % (1)	5.12 % (2)	5.12 % (2)	15.38 % (6)	71.79 % (28)	0.589
Improvement in digital model	7.69 % (3)	2.56 % (1)	2.56 % (1)	23.08 % (9)	64.11 % (25)	

## Data Availability

The data used to support the findings of this study are available from the corresponding author upon request.
